# DGGE Identification of Microorganisms Associated with *Borrelia burgdorferi* Sensu Lato- or *Anaplasma phagocytophilum*-Infected *Ixodes ricinus* Ticks from Northwest Norway

**DOI:** 10.1155/2013/805456

**Published:** 2013-10-27

**Authors:** Ann-Kristin Tveten, Andreas Riborg, Hanne Tjelle Vadseth

**Affiliations:** Department of Life Sciences, Aalesund University College, 6025 Aalesund, Norway

## Abstract

Ticks acquire a wide range of microorganisms as a natural part of their lifecycle. Bacteria, viruses, and protozoa can be transmitted to ticks during feeding and free-living phases. DGGE profiling is a molecular method to describe the microbial population associated with ticks and demonstrate some of the complexity and variety of tick-borne microorganisms. The present study profiled a total of 120 *I. ricinus* ticks, which were divided into three equally sized groups. We found that *B. burgdorferi* s.l.-infected ticks presented a pattern consisting of bacterial *Pseudomonas * spp. (67.5%), *Bacillus *spp. (50%), and *Sphingomonas *spp. (77.5%), while *A. phagocytophilum*-infected ticks were associated with *Pseudomonas *spp. (82.5%) and *Sphingomonas *spp. (57.5%). All profiles had one or more *Pseudomonas* species present, and the intramitochondrial endosymbiont Candidatus *Midichloria mitochondrii* was present in more than 25% of the samples. Statistical analysis demonstrated that the microbial communities were not significantly different between the groups and that the groups could not be characterised by a specific microbial population.

## 1. Introduction

Complex microbial communities are found in most natural ecosystems and are composed of a great variety of microorganisms [1]. Ticks have the potential to acquire microorganisms in all stages of their lifecycle, and they are associated with a large diversity of bacteria, viruses, and protozoa [2]. Some of these microorganisms are pathogens that are acquired while feeding on various hosts, while others are related to the environment where ticks reside during their free-living phases [2, 3]. Tick-borne zoonoses can cause severe and fatal infections in both humans and animals [4]. A number of tick-borne pathogens such as bacteria, viruses, and protozoa have been linked to diseases such as Lyme disease (LD), anaplasmosis (formerly ehrlichiosis), tularaemia, babesiosis, and tick-borne encephalitis virus (TBEV) [5]. 

*Borrelia burgdorferi* sensu lato comprises a group of *Borrelia* species that cause LD worldwide, and in particular, three *Borrelia *genospecies—*Borrelia burgdorferi* sensu stricto, *Borrelia afzelii*, and *Borrelia garinii*—are associated with human disease in Europe [6–8]. A fourth genospecies has been identified among Norwegian ticks: *Borrelia valaisiana* [9]. The prevalence of Lyme disease along the Norwegian coastline varies from approximately 25% in southern Norway to a prevalence of 14–18% in northwest Norway [9–11]. 

*Anaplasma phagocytophilum* causes a tick-borne rickettsial infection known as anaplasmosis [12, 13]. Human anaplasmosis is not a widespread disease in Norway [14], but tick-borne fever caused by *A. phagocytophilum* is common among livestock and has severe consequences for sheep and cattle [15]. The specific number of cases involving other tick-borne diseases such as tularaemia, babesiosis, and tick-borne encephalitis virus (TBEV) has not been reported for Norway [16]. In addition, ticks can cause polymicrobial infections due to their ability to carry multiple pathogens [17]. A synergistic effect between coinfecting microorganisms can be favourable for pathogenic microorganisms and alter their pathogenesis [18]. 

Denaturing gradient gel electrophoresis (DGGE) is a broad-range molecular method that has been utilised to determine the microbial content of ticks [19]. The method is based on broad-range amplification of 16S rDNA fragments with a GC clamp. The amplified fragments are separated in a gradient polyacrylamide gel with buffer heated to 60°C. The 16S rDNA fragments slowly melt as they migrate through the polyacrylamide gel, and the melting rate is related to the sequence composition. This makes it possible to distinguish between species, even though all 16S rDNA fragments are obtained with the same primers [20]. DGGE analyses have demonstrated that a number of bacteria and endosymbionts can be identified when analysing *I. ricinus *ticks [2, 19]. 

The aim of this study was to profile microbial communities from *I. ricinus* ticks and compare the communities associated with ticks infected by either *B. burgdorferi* sensu lato or *A. phagocytophilum* or neither of these two pathogens. We constructed two theses to be able to demonstrate whether there were significant differences in the DGGE profiles between groups and if there were significant differences in the presence of microorganisms within the DGGE profiles of each group. The present study would give indications as to whether a relationship between the presence of *B. burgdorferi* sensu lato or *A. phagocytophilum* and specific microorganisms exists. Microbial communication and interaction between microorganisms influence individual organisms differently, which makes knowledge of the total microbial community useful when studying tick-borne pathogens [21].

## 2. Materials and Methods

### 2.1. DNA Isolation from Ticks

*I. ricinus* ticks were collected from the woodlands in Skodje, a municipality in Møre and Romsdal counties, Norway, between May and October 2011. All ticks were collected by dragging a flannel cloth through the vegetation. Individual ticks (38 adult females, 442 nymphs) were placed into sterile tubes labelled with the date of collection. Due to the low number of adult ticks, the study proceeded without distinguishing between adult and nymphal ticks. Individual ticks were washed in 70% ethanol, placed individually into sterile tubes with sterile double distilled (dd) H_2_O, and homogenised using 5 mm steel beads and a Qiagen TissueLyser (Qiagen GmbH, Germany). DNA was extracted from each sample using a DNeasy blood and tissue kit (Qiagen GmbH, Germany) according to the manufacturer's protocol.

### 2.2. Detection of *B. burgdorferi* Sensu Lato and *A. phagocytophilum* Infection by Real-Time PCR (qPCR)

All tick samples were analysed by qPCR with primers specific for *B. burgdorferi* sensu lato or *A. phagocytophilum* to determine whether the ticks were infected by either of these pathogens. All primers and probes are listed in Table 1. qPCR was performed in reaction volumes of 15 *μ*L using 7.5 *μ*L of TaqMan (no UNG) Universal Master Mix (Applied Biosystems). The optimal reaction conditions contained 500 nM of each primer, 100 nM of each probe, and 3 *μ*L of template DNA. qPCR was performed in a 7300 Real-Time PCR System (Applied Biosystems) with 1 cycle of denaturation (10 min, 95°C), followed by 45 cycles of denaturation (15 sec, 95°C) and annealing/extension (1 min, 60°C).

### 2.3. 16S rDNA Amplification and Denaturing Gradient Gel Electrophoresis (DGGE)

Bacterial 16S rDNA fragments were amplified by PCR with the primers 341fGC and 518r (Table 1) numbered according to the *Escherichia coli* 16S rDNA sequence [19]. Reactions were carried out in 30 *μ*L volumes containing 15 *μ*L of Red'y'Gold PCR Master Mix (Eurogentec, Germany), 0.12 *μ*L of each primer (100 *μ*M), 9.76 *μ*L of ddH_2_O, and 5 *μ*L of template DNA. An amplification procedure modified from Schabereiter-Gurtner et al. was performed in a 2720 thermal cycler (Applied Biosystems, Carlsbad, US) with 1 cycle of denaturation (10 min, 95°C) followed by 32 cycles of denaturation (1 min, 95°C), annealing (1 min, 55°C), and extension (1 min, 72°C) along with a final cycle of extension (10 min, 72°C) [2, 19]. Separation of the amplified 16S rDNA fragments was performed with polyacrylamide gels with a linear denaturant gradient from 30% to 60%, where 100% denaturant contained 7 M urea and 40% formamide. Each well was washed with 0.5x Tris-acetate-EDTA buffer (TAE buffer) to remove casting residues, and 25 *μ*L of each amplicon was placed into one well. Denaturing gradient gel electrophoresis was performed in a V20 CDC Dual Vertical Unit System (Scie-Plas, Gainsborough Warwickshire, UK) at 60°C/20 V for 10 min and then at 60°C/60 V for 16 hr in 0.5x TAE as previously described [2]. 

### 2.4. Sequencing and Identification

16S rDNA fragments from DGGE bands were cut out from the gel and placed into tubes containing 20 *μ*L of ddH_2_O; these fragments were then used as templates for PCR using 341f/518r primers (Table 1) and the same reaction protocol as the original amplification. Purification of the amplified products was performed in a two-step process using ExoSAP-IT (Affymetrix, Santa Clara, USA) according to the manufacturer's protocol. The sequencing reactions were carried out in 10 *μ*L volumes containing 1 *μ*L of Big Dye Terminator 3.1 (Applied Biosystems, Carlsbad, USA), 1 *μ*L of 5x Sequencing Buffer (Applied Biosystems, Carlsbad, USA), 0.32 *μ*L of 341f primer (10 *μ*M), 4.68 *μ*L of ddH_2_O, and 3 *μ*L of template DNA. Amplification was performed in a 2720 Thermal Cycler (Applied Biosystems, Carlsbad, USA) with 1 cycle of denaturation (6 min, 96°C), followed by 25 cycles of denaturation (10 sec, 96°C), annealing (5 sec, 50°C), and extension (4 min, 60°C). Next, 10 *μ*L of ddH_2_O was added to each well upon completion of the sequencing reaction as previously described [2]. The source of each sequence was identified using BLAST to compare sequences from the DGGE analysis to sequences of known bacterial species in the GenBank database [24]. Sequences that exhibited less than 95% identification by BLAST search were classified by the RDP classifier [25]. 

### 2.5. Statistical Analysis

Statistical analysis was utilised to study the statistical coherence between the selection of microorganisms identified within each group and the specific pathogenic bacteria. Statistical comparison of the variance between the groupings was calculated by a one-way ANOVA analysis using IBM SPSS Statistics 20 (SPSS Inc., Chicago, USA). The significance level was set to 0.05. Analyses were performed to test whether there were significant differences between the microorganisms in *Borrelia burgdorferi* sensu lato- or *Anaplasma phagocytophilum*-infected *I. ricinus* ticks. In addition, it was determined whether there were significant differences in the presence of microorganisms within the DGGE profiles of *Borrelia burgdorferi* sensu lato- or *Anaplasma phagocytophilum-*infected *I. ricinus* ticks. Statistical analysis was performed to analyse the variance between the microbial contents from groups G1, G2, and G3. A scatter plot was created to study the distribution of bacterial species between the three groups. 

## 3. Results

### 3.1. qPCR Analysis

A total of 480 *I. ricinus* ticks were analysed by qPCR to identify *B. burgdorferi* sensu lato- or *A. phagocytophilum*-infected ticks. The results showed that 87 ticks were positive for *B. burgdorferi* sensu lato, while 47 ticks were positive for *A. phagocytophilum*. Among the positive samples, 6 samples were positive for both *B. burgdorferi* sensu lato and *A. phagocytophilum*. None of the coinfected ticks were included in further analyses. Based on the results of the qPCR analysis, three equally sized groups were designated for further analysis. The number of *A. phagocytophilum*-positive samples limited the size of each group to 40 samples. Based on this limitation, 40 *B. burgdorferi* sensu lato-positive ticks comprised group G1, 40 *A. phagocytophilum*-positive ticks comprised group G2, and 40 ticks that were not positive for either *B. burgdorferi* sensu lato or *A. phagocytophilum* comprised group G3.

### 3.2. DGGE and Sequence Identification

**Figure 1:**
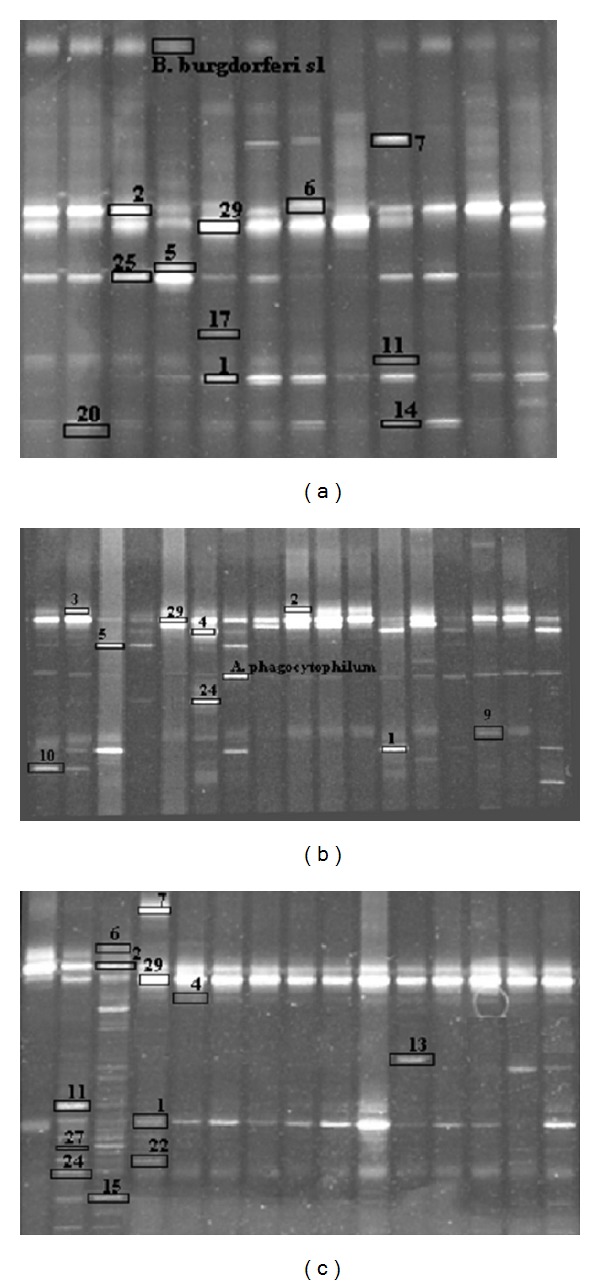
(a) SYBR Green-stained 16S rDNA DGGE profile of bacteria found in ticks from group G1. The DGGE bands have been identified in the figure with numbers corresponding to Table 2. Each lane represents one profile from a specific individual tick. Each band represents a specific bacterium. (b) SYBR Green-stained 16S rDNA DGGE profile of bacteria found in ticks from group G2. The DGGE bands have been identified in the figure with numbers corresponding to Table 2. Each lane represents one profile from a specific individual tick. Each band represents a specific bacterium. (c) SYBR Green-stained 16S rDNA DGGE profile of bacteria found in ticks from group G3. The DGGE bands have been identified in the figure with numbers corresponding to Table 2. Each lane represents one profile from a specific individual tick. Each band represents a specific bacterium.

Amplification and DGGE analysis of all 120 ticks in the three groups (G1–G3) were performed, and 120 DGGE profiles were obtained, with one for each individual tick.  Figure 1(a) shows the individual DGGE profiles (1–12) obtained from group G1, Figure 1(b) shows the individual DGGE profiles (1–17) obtained from group G2, and Figure 1(c) shows the individual DGGE profiles (1–15) obtained from group G3.

A total of 94 individual bands with different migration rates were excised, reamplified, and sequenced for identification. In addition to *B. burgdorferi* sensu lato or *A. phagocytophilum*, the sequenced strains comprised 28 different bacterial species. Of the 94 sequences, some were identified as the same species. The results from the BLAST search and RDP classification are displayed in Table 2. The *I. ricinus* 18S rDNA region was also identified within the sampled material.

Various *Pseudomonas* species were present in all samples, regardless of the group. In addition to* Pseudomonas*, the intramitochondrial endosymbiont *Candidatus Midichloria mitochondrii* and *Sphingomonas* spp. were present in a large number of ticks from groups G1 and G2. In group G1, 50% of the DGGE profiles contained *Bacillus* spp., which were present only in this group. 

### 3.3. Statistical Analysis

Statistical analyses from the data obtained through DGGE profiling were used to calculate the variance between the microbial content of all three groups. 

One-way ANOVA analysis (Table 3) gave a calculated variance ratio (*F*) of 0.630, and the critical value of significance (*F*_CRIT_) was 1.8. This indicates that the variance between the microbial content in groups G1, G2, and G3 was not significant. 

**Figure 2:**
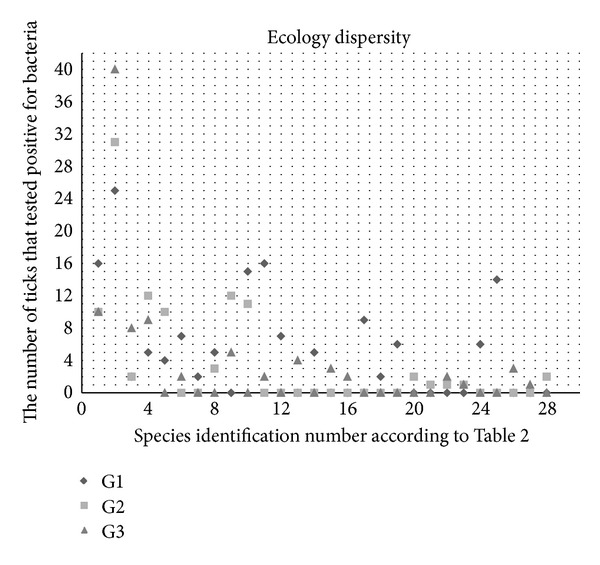
The scatter plot shows the distribution of bacterial species from groups G1, G2, and G3. Species are identified in the figure with a number corresponding to the species number in Table 2.

The results indicate that there were no significant differences between the identified microorganisms associated with *B. burgdorferi* s.l.-infected ticks, *A. phagocytophilum*-infected ticks, and ticks that were not infected by either of these two pathogens. The scatter plot (Figure 2) further demonstrates the similarities between the microbial contents from all three groups, and the dispersal of bacteria from each group is equal throughout the plot. 

The profiles of individual ticks within each group were statistically compared to calculate if there were significant differences in the presence of microorganisms within the groups G1, G2, and G3. The calculated difference within each group gave a variance ratio (*F*) of 6.905 (*F*_CRIT_ = 2.36). This indicates that there was a significant difference in microbial content within groups G1, G2, and G3 and that ticks infected by either *B. burgdorferi* sensu lato or *A. phagocytophilum* cannot be characterised by a specific DGGE profile.

## 4. Discussion

*I. ricinus* ticks are exposed to microorganisms both through feeding on various hosts and within their natural habitats [4, 26]. A wide range of bacteria, protozoa, and viruses with different pathogenic characteristics have been described as tick-borne zoonoses [3, 27]. DGGE analysis is a molecular-based tool that has been utilised to describe the tick-related microbial community beyond the known pathogenic organisms [2, 19]. Here, DGGE profiling was used to describe the microbial population in *B. burgdorferi* sensu lato- and *A. phagocytophilum*-infected ticks.

All DGGE profiles had a consistent band identified as the *I. ricinus* 18S rDNA region. The eukaryote 18S region is homologous with the bacterial 16S region, and consequently amplification of 18S fragments can occur [28]. A majority of the microorganisms identified through DGGE profiling were microorganisms that have origins from environmental samples. *Pseudomonas putida*, *Spiroplasma* spp., *Roseomonas* spp., *Methylovirgula* spp., *Methylobacterium* spp., *Erwinia billingiae*, *Raoultella* spp., and *Enterobacter* spp. were identified in the DGGE profiles from group G1, and *Roseomonas* spp., *Methylobacterium* spp., *Erwinia billingiae*, *Enterobacteriaceae*, *Stenotrophomonas* spp., *Williamsia* spp., *Uncultured Mycobacterium* spp., and *Luteibacter rhizovicinus* were present in the group G2 DGGE profiles. Bacteria commonly found in environmental samples have been demonstrated in several studies of microorganisms associated with ticks [2, 19, 29–31], and symbiotic microorganisms may play an important role in survival of the host [32–34].

Several strains of *Pseudomonas* were identified in our study, and *Pseudomonas* has frequently been detected in other studies of microbial communities in ticks [2, 19, 30, 31]. At least one species of *Pseudomonas* was identified in each tick, indicating that *Pseudomonas* may have a symbiotic association with ticks. Members of the genus *Pseudomonas* are versatile bacteria with a wide range of natural habitats [35], and it has been demonstrated that *P. fluorescens*, one of the strains identified in this study, easily forms surface biofilms [36]. *P. fluorescens* has also been associated with ticks in previous studies [2, 19]. A biofilm is a community of microorganisms attached to a surface, and microorganisms located within a biofilm matrix are difficult to remove [36]. Bacteria-host interactions through a biofilm matrix can be beneficial for both the bacteria and the host and can increase the host's ability to survive [21, 32–34]. A study by Carpi et al. identified bacteria commonly found in soil samples, although a rigorous washing procedure was performed, and suggested that these bacteria were a part of the tick exoskeleton [37]. A biofilm matrix or microorganisms incorporated into the exoskeleton might explain why various environmentally associated bacteria could be identified amongst the tick samples in this study. 

Other than the known tick-borne pathogens *B. burgdorferi* sensu lato or *A. phagocytophilum*, none of the well-known tick-borne pathogens was detected. Studies of *I. ricinus* ticks from Europe have detected several pathogenic species such as *Rickettsia*, *Babesia*, *Bartonella*, and *Candidatus Neoehrlichia mikurensis* [38–41]. While *Rickettsiella *spp. and a *Rickettsiales* bacterium were detected in a previous study of *I. ricinus* ticks from northwest Norway [2], these were not identified in the present study. The overall prevalence of *Borrelia* genospecies varies between 14,8% and 18.7% [11], but the prevalence of most tick-borne pathogens in the fauna in northwest Norway has not yet been examined. 

Statistical analyses were performed to further describe the variance between the microbial communities from each grouping. The scatter plot illustrates the dispersion of bacterial species and the similarity between the microbial communities. The one-way ANOVA analysis indicates that there is no statistically significant variance between the microorganisms identified in G1, G2, and G3. This indicates that the presence or absence of any microorganism in ticks does not seem to be related to a specific pathogen. 

Group G1 consisted of *I. ricinus *ticks infected by spirochetes of the *B. burgdorferi *sensu lato group, which is the etiological agent of Lyme borreliosis. In group G1, *Pseudomonas *spp., *Bacillus *spp*., *and *Sphingomonas *spp., were present in a majority of the DGGE profiles.* Pseudomonas *spp. and *Bacillus* spp. have previously been identified from cultured bacterial flora from *I. ricinus *ticks [31]. The two most frequent profiles from group G1 contained a combination of the bacteria *Pseudomonas* spp. and *Bacillus* spp. or *Pseudomonas* spp. and *Sphingomonas* spp. Calculation of statistical variance within G1 indicates that this group cannot be characterised by one specific microbial profile. 

Group G2 consisted of *I. ricinus *ticks infected by *A. phagocytophilum*. During an *A. phagocytophilum* infection, the obligate intracellular *A. phagocytophilum* bacterium targets myeloid or granulocytic cells to propagate. The bacterium divides, and cell lysis releases bacteria that can infect other cells [13]. Several *Pseudomonas* species and *Sphingomonas* spp. were among the recurring bacterial types that constituted the majority of the microorganisms identified in *A. phagocytophilum*-infected ticks. The two most frequent profiles from group G2 contained a combination of the bacteria *Pseudomonas* spp. and *Pseudomonas fluorescens* or *Pseudomonas* spp. and *Sphingomonas* spp. Calculation of statistical variance within G2 indicates significant differences between the profiles within group G2, and no specific profile can be used to describe ticks infected by *A. phagocytophilum*.

Group G3 included *I. ricinus *ticks that were not infected by either *B. burgdorferi* sensu lato or *A. phagocytophilum*. DGGE profiles from this group contained one or more species of *Pseudomonas*. In addition to the endosymbiont *Candidatus Midichloria mitochondrii* and the band from the *I. ricinus* 18S rDNA region, each profile generally contained 1-2 bacterial species. *Sphingomonas* spp., *Pseudomonas putida, Methylobacterium* spp., and *Burkholderia* spp., *Erwinia billingiae*, *Williamsia* spp., and uncultured *Mycobacterium* spp., *Mycobacterium, Beijerinckiaceae* constituted the bacteria identified in group G3. The most frequent profile in group G3 was a combination of two different *Pseudomonas *species. Statistically, there are no specific microbial profiles that can be used to describe group G3. The variance ratio calculated for species within the microbial communities confirmed that no specific profile could be identified within each group. 

DGGE profiling was a useful tool to demonstrate the microbial populations associated with *B. burgdorferi* sensu lato- and *A. phagocytophilum*-infected ticks. Groups G1–G3 could not be characterised by a specific microbial population, and statistical analyses confirmed that there were no significant differences in the microbial diversity for the different groups. These findings indicate that a specific pathogen, such as *B. burgdorferi* sensu lato and *A. phagocytophilum*, does not affect the diversity of the microbial content in ticks. The random pattern of microorganisms associated with ticks may indicate that microorganisms are acquired independently from each other. Bacteria associated with soil and environmental samples, in particular *Pseudomonas* species, were present in all samples. The high prevalence of *Pseudomonas* may indicate a symbiotic relationship between bacteria and vector host. The present study has also presented an overview of the microbial population associated with *I. ricinus* ticks and demonstrated a diverse microbial content associated with ticks, thus allowing a better understanding of the selection pressure from the microbial content associated with ticks. 

## Figures and Tables

**Table 1:** List of DGGE primers, qPCR primers, and probes in 5′ to 3′ orientation.

Target species	Name	Sequence	Ref.
*Anaplasma phagocytophilum*	ApMSP2f	ATGGAAGGTAGTGTTGGTTATGGTATT	
	ApMSP2r	TTGGTCTTGAAGCGCTCGTA	[22]
	ApMSP2p	TGGTGCCAGGGTTGAGCTTGAGATTG	

*Borrelia burgdorferi* sensu lato	recAf recAr	GTGGATCTATTGTATTAGATGAAGCTCTTG GCCAAAGTTCTGAAACATTAACTCCCAAAG	[23]
	BbslP	ATAGCTGCTTTTATTGATGCTGA	This study

Multispecies	341f	CCTACGGGAGGCAGCAG	
	341fGC	CGCCCGCCGCGCGCGGCGGGCGGGGCGGGG GCACGGGGGGCCTACGGGAGGCAGCAG	[19]
	518r	ATTACCGCGGCTGCTGG	

**Table 2:** Identification of bacteria associated with ticks obtained by sequence analysis of reamplified DGGE bands.

Species number^b^	Closest related sequence^a^	Percent similarity	GenBank ID	*B. burgdorferi *sensu lato-infected ticks		*A. phagocytophilum*-infected ticks		Ticks not infected by *B. burgdorferi *sensu lato or *A. phagocytophilum *	
				G1 (*n* (*N*))^c^	%	G2 (*n* (*N*))	%	G3 (*n* (*N*))	%
1	*Candidatus Midichloria mitochondrii *	100%	CP002130.0	16 (40)	40	10 (40)	25	10 (40)	25
2	*Pseudomonas *spp.	100%	JQ598792.1	25 (40)	62,5	31 (40)	77,5	40 (40)	100
3	*Pseudomonas *spp.	100%	JN630834	2 (40)	5	2 (40)	5	8 (40)	20
4	*Pseudomonas fluorescens *	100%	JX131014.1	5 (40)	12,5	12 (40)	30	9 (40)	22,5
5		100%	EF428995.1	4 (40)	10	10 (40)	25	0 (40)	0
6	*Pseudomonas putida *	99%	HQ18860l.1	7 (40)	17,5	0 (40)	0	2 (40)	5
7	*Spiroplasma *spp.	100%	AJ132412.1	2 (40)	5	0 (40)	0	0 (40)	0
8	*Roseomonas *spp.	100%	DQ532254	5 (40)	12,5	3 (40)	7,5	0 (40)	0
9	*Sphingomonas *spp.	100%	NR044341	0 (40)	0	12 (40)	30	5 (40)	12,5
10	*Sphingomonas *spp.	100%	JQ660272.1	15 (40)	37,5	11 (40)	27,5	0 (40)	0
11	*Sphingomonas *spp.	<95%		16 (40)	40	0 (40)	0	2 (40)	5
12	*Methylovirgula *spp.	99%	FM252035.1	7 (40)	17,5	0 (40)	0	0 (40)	0
13	*Methylobacterium* spp.	100%	JQ617889.1	0 (40)	0	0 (40)	0	4 (40)	10
14		<95%		5 (40)	12,5	0 (40)	0	0 (40)	0
15	*Burkholderia *spp.	100%	FN298915.1	0 (40)	0	0 (40)	0	3 (40)	7,5
16	*Erwinia billingiae *	100%	FP236843.1	0 (40)	0	0 (40)	0	2 (40)	5
17	*Erwinia billingiae *	100%	AM117487.1	9 (40)	22,5	0 (40)	0	0 (40)	0
18	*Raoultella *spp.	100%	AY292873	2 (40)	5	0 (40)	0	0 (40)	0
19	*Enterobacter *spp.	100%	JN850605.1	6 (40)	15	0 (40)	0	0 (40)	0
20	*Enterobacteriaceae *	99%	JX162048.1	0 (40)	0	2 (40)	5	0 (40)	0
21	*Stenotrophomonas rhizophila *	100%	JX005877.1	0 (40)	0	1 (40)	2,5	0 (40)	0
22	*Williamsia *spp.	100%	FN550136.1	0 (40)	0	1 (40)	2,5	2 (40)	5
23	*Uncultured Mycobacterium *spp.	100%	GQ203456.1	0 (40)	0	1 (40)	2,5	1 (40)	2,5
24	*Bacillus *spp.	100%	NR042286.1	6 (40)	15	0 (40)	0	0 (40)	0
25	*Bacillus *spp.	100%	EU647705	14 (40)	35	0 (40)		0 (40)	0
26	*Mycobacterium *	<95%		0 (40)	0	0 (40)	0	3 (40)	7,5
27	*Beijerinckiaceae *	<95%		0 (40)	0	0 (40)	0	1 (40)	2,5
28	*Luteibacter rhizovicinus *	100%	AB627008	0 (40)	0	2 (40)	5	0 (40)	0

29	*I. ricinus* 18S rDNA	100%							

^a^The source of each sequence was identified using BLAST to compare sequences from the DGGE analysis to sequences of known bacterial species in the GenBank database [24]. Sequences that exhibited less than 95% identification by BLAST search were classified by the RDP classifier [25].

^b^Species number corresponds with the numbering of bands on the DGGE gel as shown in Figures 1(a), 1(b), 1(c), and 2.

^c^ *n* (*N*)  *n*: number of positive ticks in this group, (*N*): total number of ticks analysed in this group.

**Table 3:** One-way ANOVA analysis of the coherence between groupings of microbial communities and between species.

One-way ANOVA					
Coherence between groups G1, G2, and G3					
	Sum of squares	df	Mean square	*F*	Sig.
Between groups	62.571	2	31.286	.630	.535
Within groups	4021.429	81	49.647		

Total	4084.000	83			
